# High-dose alkylating chemotherapy in BRCA-altered triple-negative breast cancer: the randomized phase III NeoTN trial

**DOI:** 10.1038/s41523-023-00580-9

**Published:** 2023-09-09

**Authors:** Sonja Vliek, Florentine S. Hilbers, Erik van Werkhoven, Ingrid Mandjes, Rob Kessels, Sieta Kleiterp, Esther H. Lips, Lennart Mulder, Mutamba T. Kayembe, Claudette E. Loo, Nicola S. Russell, Marie-Jeanne T. F. D. Vrancken Peeters, Marjo J. Holtkamp, Margaret Schot, Joke W. Baars, Aafke H. Honkoop, Annelie J. E. Vulink, Alex L. T. Imholz, Suzan Vrijaldenhoven, Franchette W. P. J. van den Berkmortel, Jetske M. Meerum Terwogt, Jolanda G. Schrama, Philomeen Kuijer, Judith R. Kroep, Annemieke van der Padt-Pruijsten, Jelle Wesseling, Gabe S. Sonke, Kenneth G. A. Gilhuijs, Agnes Jager, Petra Nederlof, Sabine C. Linn

**Affiliations:** 1 Department of Molecular Pathology, The Netherlands Cancer Institute, Amsterdam, The Netherlands; 2 Department of Medical Oncology, University Medical Center Utrecht, Utrecht, The Netherlands; 3 Department of Biometrics, The Netherlands Cancer Institute, Amsterdam, The Netherlands; 4 HOVON Data Center, Erasmus MC Cancer Institute, Rotterdam, The Netherlands; 5 Department of Radiology, The Netherlands Cancer Institute, Amsterdam, The Netherlands; 6 Department of Radiation Oncology, The Netherlands Cancer Institute, Amsterdam, The Netherlands; 7 Department of Surgical Oncology, The Netherlands Cancer Institute, Amsterdam, The Netherlands; 8 Department of Surgery, Amsterdam University Medical center, Amsterdam, The Netherlands; 9 Department of Medical Oncology, The Netherlands Cancer Institute, Amsterdam, The Netherlands; 10 Department of Internal Medicine, Isala Klinieken, Zwolle, The Netherlands; 11 Division of Medical Oncology, Reinier de Graaf Hospital, Delft, The Netherlands; 12 Department of Internal Medicine, Deventer Ziekenhuis, Deventer, The Netherlands; 13 Department of Internal Medicine, Northwest Clinics, Alkmaar, The Netherlands; 14 Department of Oncology, Zuyderland Medisch Centrum, Heerlen-Geleen, The Netherlands; 15 Department of Internal Medicine, Onze Lieve Vrouwe Gasthuis, Amsterdam, The Netherlands; 16 Department of Internal Medicine, Spaarne Gasthuis, Hoofddorp, The Netherlands; 17 Department of Medical Oncology, Leiden University Medical Center, Leiden, The Netherlands; 18 Department of Internal Medicine, Maasstad Hospital, Rotterdam, The Netherlands; 19 Department of Pathology, The Netherlands Cancer Institute, Amsterdam, The Netherlands; 20 Image Sciences Institute, University Medical Center Utrecht, Utrecht, Netherlands; 21 Department of Medical Oncology, Erasmus MC Cancer Institute, Rotterdam, The Netherlands; 22 Department of Molecular diagnostics, The Netherlands Cancer Institute, Amsterdam, The Netherlands; 23 Department of Pathology, University Medical Center Utrecht, Utrecht, The Netherlands

**Keywords:** Breast cancer, Chemotherapy

## Abstract

Exploratory analyses of high-dose alkylating chemotherapy trials have suggested that BRCA1 or BRCA2-pathway altered (BRCA-altered) breast cancer might be particularly sensitive to this type of treatment. In this study, patients with BRCA-altered tumors who had received three initial courses of dose-dense doxorubicin and cyclophosphamide (ddAC), were randomized between a fourth ddAC course followed by high-dose carboplatin-thiotepa-cyclophosphamide or conventional chemotherapy (initially ddAC only or ddAC-capecitabine/decetaxel [CD] depending on MRI response, after amendment ddAC-carboplatin/paclitaxel [CP] for everyone). The primary endpoint was the neoadjuvant response index (NRI). Secondary endpoints included recurrence-free survival (RFS) and overall survival (OS). In total, 122 patients were randomized. No difference in NRI-score distribution (*p* = 0.41) was found. A statistically non-significant RFS difference was found (HR 0.54; 95% CI 0.23–1.25; *p* = 0.15). Exploratory RFS analyses showed benefit in stage III (*n* = 35; HR 0.16; 95% CI 0.03–0.75), but not stage II (*n* = 86; HR 1.00; 95% CI 0.30–3.30) patients. For stage III, 4-year RFS was 46% (95% CI 24–87%), 71% (95% CI 48–100%) and 88% (95% CI 74–100%), for ddAC/ddAC-CD, ddAC-CP and high-dose chemotherapy, respectively. No significant differences were found between high-dose and conventional chemotherapy in stage II-III, triple-negative, BRCA-altered breast cancer patients. Further research is needed to establish if there are patients with stage III, triple negative BRCA-altered breast cancer for whom outcomes can be improved with high-dose alkylating chemotherapy or whether the current standard neoadjuvant therapy including carboplatin and an immune checkpoint inhibitor is sufficient. *Trial Registration:* NCT01057069.

## Introduction

Although the prognosis for breast cancer patients has improved substantially over the last decades, breast cancer remains responsible for ~600,000 deaths each year worldwide^1^. Therefore, there is a persisting need to find better treatment options for breast cancer patients at high risk of disease recurrence and death. High-dose chemotherapy with autologous stem-cell support has been investigated as treatment strategy for high-risk, early breast cancer patients. A meta-analysis reported a statistically significant, 13% relative reduction in disease recurrence for patients treated with high-dose chemotherapy, while the effect on overall survival (OS) remained non-significant^2^. However, chemotherapy regimens given in these clinical trials were very heterogeneous. In some trials the total chemotherapy dose-intensity was higher in the control arm than in the high-dose arm. When the summation dose intensity (SDI), a measure of average weekly dose-intensity^3^, was used, a significant improvement in both recurrence-free survival (RFS) and OS was found for every 0.05 unit of increase in SDI^2^. Especially in the triple-negative breast cancer (TNBC) subgroup a substantial high-dose chemotherapy benefit was observed^2^. However, the substantial toxicity associated with high-dose chemotherapy^4^ makes that there is currently no clearly defined patient population for which this treatment strategy is considered appropriate.

A post-hoc analysis of a large trial (the N4+ study, NCT03087409) has found a much larger reduction in disease recurrence by high-dose, alkylating chemotherapy for the subgroup of stage III, HER2-negative breast cancer patients with so-called BRCA1-like tumors (hazard rate (HR) 0.12) compared to non-BRCA1-like tumors (HR 0.78)^5^. These data have subsequently been confirmed in other retrospective analyses^6,7^. BRCA1-like tumors are characterized by a specific genome-wide pattern of copy number aberrations that is indicative of the absence of a functional BRCA1 protein, either through bi-allelic *BRCA1* mutation (or loss of heterozygosity), *BRCA1* promoter methylation or through an as yet unidentified mechanism^8,9^. The absence of functional BRCA1 results in homologous recombination deficiency and therefore the inability to repair DNA double strand breaks in an error-free manner^10^. As high-dose, alkylating chemotherapy induces DNA double strand breaks, an increased sensitivity to this treatment can be explained by the absence of functional BRCA1 and a similar effect would be expected for tumors with mutations in other homologous recombination repair genes such as *BRCA2*.

The current randomized study aims to provide prospective data on the efficacy of high-dose alkylating chemotherapy in patients with stage II or III, BRCA1-, or BRCA2-pathway altered (BRCA-altered) TNBC to contribute to a better definition of the patient population for which high-dose chemotherapy would be an appropriate treatment strategy.

## Results

### Patient characteristics

**Fig. 1:**
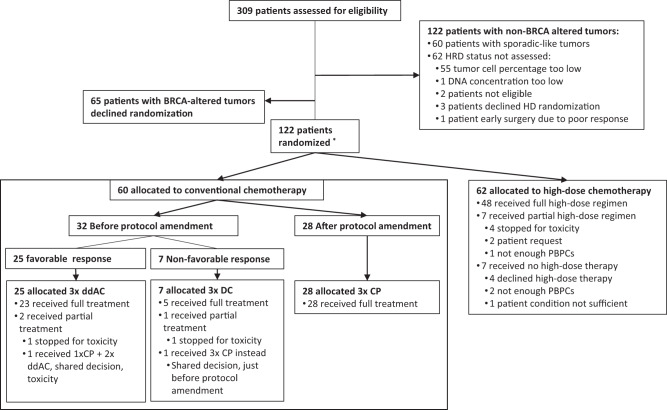
CONSORT diagram. *Three ineligible patients were randomized by mistake: two patients with a tumor that showed a sporadic-like profile, but that at the same time was positive for *BRCA1* promoter hyper-methylation were randomized to the conventional chemotherapy arm. One received ddAC-only, the other ddAC-CP. One patient was found to be metastatic at baseline and was randomized to the high-dose arm. These patients remained part of the ITT analysis.

Between February 2010 and February 2016, 309 patients enrolled in the NeoTN trial (see Fig. 1). Out of these patients, 187 (60%) had a tumor that was classified as BRCA-altered of whom 65 (35%) declined randomization between high-dose and conventional chemotherapy. Of the 122 randomized patients, 60 were allocated to conventional and 62 to high-dose chemotherapy. Within the conventional treatment arm, 7 (12%), 25 (42%) and 28 (47%) patients received ddAC-CD, ddAC-only and ddAC-CP, respectively. A detailed breakdown of BRCA1-like status, BRCA1 promoter hypermethylation status and germline mutations can be found in Supplementary Tables 1 and 2.

Table 1 shows the patient characteristics for the 122 patients randomized between conventional and high-dose neoadjuvant chemotherapy (Supplementary Table 3 gives a breakdown by conventional chemotherapy regimens). Most patients had stage II disease: i.e. 70% for the conventional and 71% high-dose arm. Overall, the arms were well balanced, but slightly more patients in the conventional arm were diagnosed with node-negative disease (43% vs 34%) and with a poorly differentiated tumor (92% vs 78%) compared to the high-dose arm. The MRI-response to the first three ddAC courses was similar in both arms, with 83% and 84% showing a favorable response in the conventional and high-dose arm, respectively. Slightly more patients in the high-dose arm underwent breast conserving surgery (58% vs 47%) and received radiotherapy (84% vs 73%). Only 10 (17%) patients in the conventional and 3 (5%) patients in the high-dose arm received any adjuvant chemotherapy (all these patients did not achieve a pCR).

**Table 1:** Patient characteristics.

	Conventional chemotherapy *n* = 60 (%)	High-dose chemotherapy *n* = 62 (%)	All randomized patients *n* = 122 (%)
Median age (IQR)	42 (37–51)	43 (36–50)	43 (37–51)
Menopausal status^a^			
Pre	41 (68%)	43 (70%)	84 (69%)
Peri	5 (8%)	6 (10%)	11 (9%)
Post	14 (23%)	12 (20%)	26 (21%)
NA	0	1	1
Size (T)			
1	4 (7%)	4 (6%)	8 (7%)
2	44 (73%)	47 (76%)	91 (75%)
3	10 (17%)	10 (16%)	20 (16%)
4	2 (3%)	1 (2%)	3 (2%)
Nodal status (N)			
0	26 (43%)	21 (34%)	47 (39%)
1	20 (33%)	26 (42%)	46 (38%)
2	4 (7%)	5 (8%)	9 (7%)
3	10 (17%)	10 (16%)	20 (16%)
Stage			
II	42 (70%)	44 (71%)	86 (70%)
III	18 (30%)	17 (27%)	35 (29%)
IV	0 (0%)	1 (2%)	1 (1%)
Grade			
Well differentiated	0 (0%)	1 (2%)	1 (1%)
Moderately differentiated	3 (8%)	10 (20%)	13 (15%)
Poorly differentiated	34 (92%)	38 (78%)	72 (84%)
NA	23	13	36
Copy number profile			
BRCA1-like	51 (96%)	57 (98%)	108 (97%)
Sporadic^b^	2 (4%)	1 (2%)	3 (3%)
NA^b^	7	4	11
Germline mutation			
*BRCA1*	12 (27%)	12 (25%)	24 (26%)
*BRCA2*	0 (0%)	2 (4%)	2 (2%)
*PALB2*	1 (2%)	0 (0%)	1 (1%)
No^c^	32 (71%)	34 (71%)	66 (71%)
NA^d^	15	14	29
BRCA1 promoter hypermethylation			
Yes	17 (46%)	13 (34%)	30 (41%)
No	20 (54%)	25 (66%)	44 (59%)
NA^e^	23	24	47
MRI response^f^			
Favorable	50 (83%)	52 (87%)	102 (84%)
Non-favorable	10 (17%)	8 (13%)	18 (15%)
NA	0	2	2
Breast conserving surgery	28 (47%)	36 (58%)	64 (52%)
Radiotherapy	44 (73%)	52 (84%)	96 (79%)

^a^As assessed by the treating physician, LH, FSH and 17-beta-estradiol were tested in case of doubt.

^b^Patients with a sporadic profile or for whom no copy number profile could be obtained have been included in the HRD population of this trial based on a *BRCA1* or *BRCA2* germline mutation or *BRCA1* promotor methylation.

^c^During the conduct of this trial, germline genetics testing typically only included *BRCA1* and *BRCA2*.

^d^Local guidelines recommended referral for germline genetics testing for women diagnosed with triple-negative breast cancer before the age of 50. Germline mutation status was updated during follow-up if new information became available.

^e^BRCA1 promoter hypermethylation testing was only mandatory if results for the BRCA1-like test could not be obtained and if the patient did not have a *BRCA1* or *BRCA2* germline mutation. In other cases, BRCA1 promoter hypermethylation testing depended on the amount of remaining tissue from the biopsy.

^f^After the first 3 cycles of neoadjuvant ddAC.

### Locoregional response

**Fig. 2:**
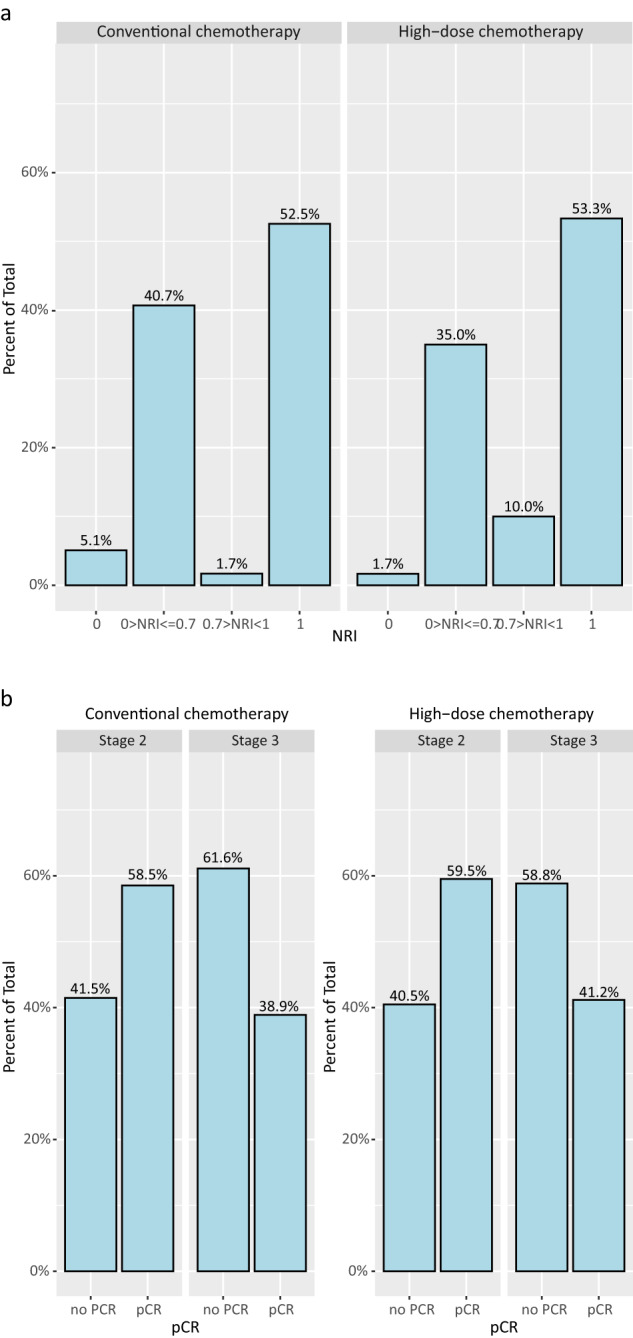
Locoregional response. **a** Neoadjuvant Response Index (NRI) by chemotherapy arm, **b** pathological complete response (pCR) rate by disease stage, stratified by treatment arm.

Figure 2a shows the NRI scores in the two treatment arms. The distribution of scores was very similar in the conventional and high-dose arm, although slightly more patients in the high-dose arm had an NRI score larger than 0.7. The average NRI score was 0.72 in the conventional and 0.78 in the high-dose arm (*p* = 0.41). In the conventional arm 31 (52.5%) patients achieved a pCR, compared to 32 (53.3%) in the high-dose arm. Of note, although fewer stage III than stage II patients achieved a pCR, there was no difference within these groups based on treatment (Fig. 2b).

### Safety

Table 2 provides an overview of the most common, non-hematological, AEs in high-dose and conventional chemotherapy arms (excluding AEs during the first three ddAC courses). No treatment related deaths occurred in either treatment arm. High-dose chemotherapy was more often associated with fatigue (grade ≥2 89% vs 39%; grade 3–4 40% vs 4%), infections (including febrile neutropenia, grade ≥2 62% vs 12%; grade 3–4 55% vs 3%), and gastrointestinal AEs such as nausea (grade ≥2 75% vs 10%; grade 3–4 25% vs 1%), oral mucositis (grade ≥2 47% vs 15%; grade 3–4 19% vs 4%) and diarrhea (grade ≥2 43% vs 4%; grade 3–4 13% vs 0%). The median time between the start of the last chemotherapy course and surgery was slightly longer in the high-dose (40 days, IQR 35–44) compared to the conventional arm (33 days, IQR 26–40). For the 74 patients treated at the Netherlands Cancer Institute, additional information was collected on complications related to surgery and radiotherapy. Complications were reported in 2/33 (6%) and 4/39 (10%) patients in the conventional and high-dose arm respectively (Supplementary Table 4). During follow-up, two patients developed a second primary cancer, one in situ melanoma and one contralateral breast cancer, both in the conventional chemotherapy arm.

**Table 2:** Adverse events.

		ddAC	ddAC-CD	ddAC-CP	All conventional	High-dose
	Grade	*n* = 24	*n* = 7	*n* = 36	*n* = 67	*n* = 53
Fatigue	**Any**	12 (50%)	3 (43%)	11 (31%)	26 (39%)	47 (89%)
	**3**–**4**	2 (8%)	1 (14%)	0 (0%)	3 (4%)	21 (40%)
Nausea	**Any**	5 (21%)	0 (0%)	2 (6%)	7 (10%)	40 (75%)
	**3**–**4**	1 (4%)	0 (0%)	0 (0%)	1 (1%)	13 (25%)
Infection^a^	**Any**	3 (13%)	2 (29%)	3 (8%)	8 (12%)	33 (62%)
	**3**–**4**	0 (0%)	1 (14%)	1 (3%)	2 (3%)	29 (55%)
Mucositis oral	**Any**	7 (29%)	2 (29%)	1 (3%)	10 (15%)	25 (47%)
	**3**–**4**	2 (8%)	1 (14%)	0 (0%)	3 (4%)	10 (19%)
Diarrhea	**Any**	1 (4%)	2 (29%)	0 (0%)	3 (4%)	23 (43%)
	**3**–**4**	0 (0%)	0 (0%)	0 (0%)	0 (0%)	7 (13%)
Vomiting	**Any**	2 (8%)	0 (0%)	0 (0%)	2 (3%)	22 (42%)
	**3**–**4**	0 (0%)	0 (0%)	0 (0%)	0 (0%)	6 (11%)
Allergic reaction	**Any**	0 (0%)	1 (14%)	0 (0%)	1 (1%)	19 (36%)
	**3**–**4**	0 (0%)	0 (0%)	0 (0%)	0 (0%)	3 (6%)
Skin related adverse events^b^	**Any**	2 (8%)	6 (86%)	1 (3%)	9 (13%)	10 (19%)
	**3**–**4**	0 (0%)	2 (29%)	1 (3%)	3 (4%)	1 (2%)
Pain	**Any**	0 (0%)	0 (0%)	1 (3%)	1 (1%)	14 (26%)
	**3**–**4**	0 (0%)	0 (0%)	0 (0%)	0 (0%)	4 (8%)
Gastritis, reflux or dyspepsia^c^	**Any**	2 (8%)	0 (0%)	1 (3%)	3 (4%)	10 (19%)
	**3**–**4**	0 (0%)	0 (0%)	0 (0%)	0 (0%)	2 (4%)
Peripheral sensory neuropathy	**Any**	2 (8%)	3 (43%)	5 (14%)	10 (15%)	1 (2%)
	**3**–**4**	1 (4%)	0 (0%)	1 (3%)	2 (3%)	0 (0%)
Weight loss	**Any**	3 (13%)	0 (0%)	0 (0%)	3 (4%)	6 (12%)
	**3**–**4**	0 (0%)	0 (0%)	0 (0%)	0 (0%)	0 (0%)

Excluding the first three cycles of ddAC. Grade 1 adverse events were not registered. All non -hematological adverse events with a frequency for grade 2–4 of at least 10% in either the conventional or high-dose treatment arm are listed. ^a^combined all infection CTCAE terms and includes febrile neutropenia.

^b^combined CTCAE “rash”, “palmar-plantar erythrodysesthesia syndrome” and “pruritus”,.

^c^combined CTCAE “gastritis”, “gastroesophageal reflux disease” and “dyspepsia”.

### Recurrence-free survival

**Fig. 3:**
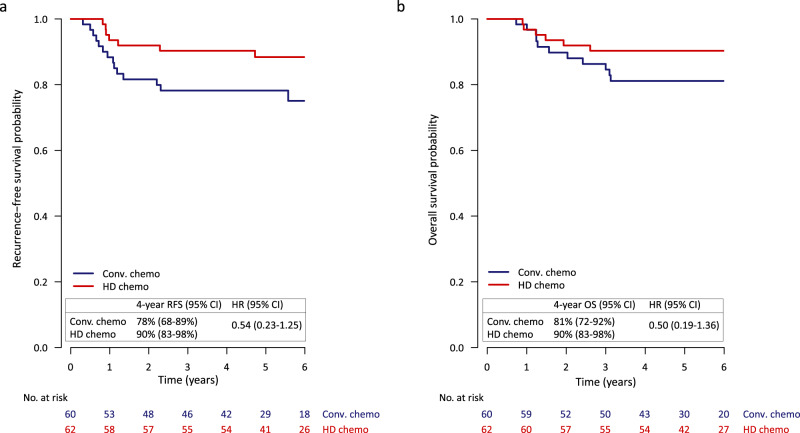
Recurrence-free and overall survival. **a** Recurrence-free survival (RFS) by treatment arm and **b** Overall survival by treatment arm for high-dose (HD) chemotherapy vs. conventional (Conv.) chemotherapy.

**Fig. 4:**
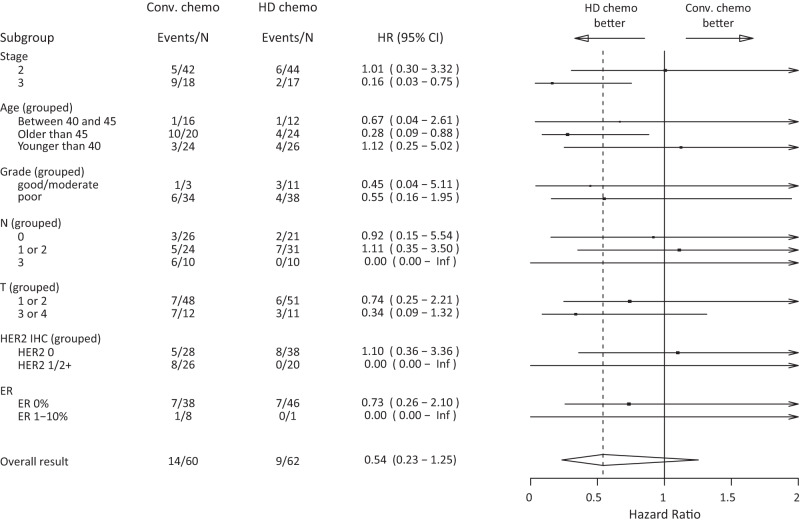
Forest plot recurrence-free survival. Recurrence-free survival hazard ratios (HR) for high-dose (HD) chemotherapy vs. conventional (Conv.) chemotherapy for various patient subgroups.

The median follow-up was 5.7 years (IQR 4.7–7.2 years). Figure 3A shows the Kaplan-Meier curves for the RFS in the two ITT treatment arms. The estimated 4-year RFS was 78% (95% CI 68–89%) for the conventional arm and 90% (95% CI 83–98%) for the high-dose arm respectively, resulting in a HR of 0.54 (95% CI 0.23–1.25). When stratifying RFS by stage, we observed a notable difference in the benefit from high-dose over conventional therapy, with a HR of 1.00 (95% CI 0.30–3.30) for stage II and a HR of 0.16 (95% CI 0.03–0.75) for stage III disease (see Supplementary Figure 1). In stage III disease, the conventional arm under the original protocol (ddAC only or ddAC-CD depending on initial MRI response) had a 4-year RFS of 46% (95% CI 24–87%), compared to 71% (95% CI 48–100%) under the protocol amendment (ddAC-CP) and 88% (74–100%) for the high-dose arm (Supplementary Fig. 2). No other subgroups showed a clear differential high-dose chemotherapy benefit (Fig. 4).

Given the similar pCR rates in both treatment arms, we next assessed how achieving pCR affected RFS^surgery^ (Supplementary Fig. 3). In the conventional chemotherapy arm, the 4-year RFS^surgery^ was 94% (95% CI 85–100%) and 60% (95% CI 44–82%) for patients who did achieved a pCR and those who did not, respectively. Interestingly, both stage II and stage III patients who achieved a pCR in the high-dose arm had a 4-year RFS^surgery^ of 100% (95% CI NA), compared to 82% (95% CI 66–100%) in stage II and 80% (95% CI 59–100%) in stage III patients who did not achieve a pCR. Also, NRI was a strong predictor for RFS^surgery^. In a multivariable model including stage and NRI (as continuous variable), we found a HR of 0.04 (95% CI 0.01–0.16) for NRI, meaning that for every 0.1 increase in NRI, the risk of a RFS event was 27.6% lower. Even when excluding patients who achieved a pCR (NRI = 1), the predictive value of NRI remained (HR 0.08, 95% CI 0.01–0.56).

### Overall survival

The 4-year OS was 81% (95% CI 72–92%) in the conventional arm compared to 90% (95% CI 83–98%) in the high-dose arm (Fig. 3B), resulting in a HR of 0.50 (95% CI 0.19–1.36, *p* = 0.17) for high-dose versus conventional therapy. For OS, similar to RFS, a larger difference between the arms was observed for stage III patients, with a 4-year OS of 64% (95% CI 44–92%) for conventional and 88% (95% CI 74–100%) for high-dose chemotherapy, compared to stage II with 88% (95% CI 79–99%) and 93% (95% CI 86–100%) for conventional and high-dose chemotherapy, respectively (Supplementary Fig. 4). For OS, however, the interaction between stage and treatment was not statistically significant (*p* = 0.45 in a multivariable analysis). Although we observe less distant recurrences in the high-dose arm (*n* = 6) compared to the conventional chemotherapy arm (*n* = 12), patients in the high-dose arm seem to do worse than those in the conventional arm after distant recurrence, with a 6-month survival probability of 33% (95% CI 11–100%) and 64% (95% CI 41–100%), respectively (Supplementary Fig. 5).

## Discussion

In this prospective, randomized clinical trial comparing alkylating high-dose and conventional neoadjuvant chemotherapy in women with BRCA-altered breast cancer, the distribution of NRI scores was not significantly different between the arms. Likewise, no difference in pCR was found. Interestingly, although not statistically significant, a difference in 4-year RFS was found (78% [95% CI 68–89%] vs 90% [95% CI 83–98%]), between conventional and high-dose chemotherapy. Of note, as shown previously in other cohorts^11,12^, this study finds that the NRI is strongly predictive for RFS even when excluding cases who achieve pCR (NRI = 1), indicating prognostic value beyond that of pCR.

Previous randomized clinical trials with high-dose chemotherapy have mostly included stage III patients. Therefore, the efficacy of this treatment in stage II patients was unclear and there is no data on BRCA-altered status as a biomarker for high-dose chemotherapy benefit in these patients. As patients with stage II disease already have a relatively good prognosis, giving these patients high-dose chemotherapy might be overtreatment. We examined treatment benefit in stage II and III separately and found that only stage III patients seem to benefit from high-dose chemotherapy (RFS HR 0.16 [95% CI 0.03–0.75] compared to HR 1.00 [95% CI 0.30–3.30] for stage II). To our knowledge this is the first study to assess the possible interaction between treatment and stage for high-dose chemotherapy. Studies stratifying treatment benefit by the number of positive lymph nodes have found conflicting results^2,13^, which might be due to the heterogeneity in high-dose regimens. Our results for stage III disease are in line with the results of the post-hoc analysis of the N4+ study, which found a HR of 0.12 and only included stage III patients^5^. As the meta-analysis by Berry et al. found a worse survival after recurrence for the high-dose arm^2^, we also assessed OS after distant recurrence in our population and found a similar effect. However, the number of distant recurrences in the high-dose arm is lower compared to the conventional arm (6 vs 12). The shorter survival after distant recurrence might be due to only very aggressive and fully chemotherapy-resistant cancer cells being able to survive high-dose chemotherapy.

Our study has a number of limitations. During the conduct of this trial, results from the GeparSixto and CALGB-40603 became available which showed that adding neoadjuvant carboplatin improves the pCR rate in TNBC^14,15^. This increased pCR rate associated with carboplatin use was later also confirmed by the BrighTNess study^16^. Based on these results, we amended the study protocol such that all patients randomized to the conventional arm received three courses of CP after the initial three ddAC courses. In the end, 28/60 patients in the conventional arm received this regimen. This gave us only very limited power to compare high-dose chemotherapy and carboplatin-containing conventional chemotherapy. However, exploratory analysis suggested that the 4-year RFS in the stage III patients treated with CP is still slightly lower than that in the high-dose arm. The power of our study was also affected by the fact that the sample size calculation had not anticipated a substantial drop-out of patients in the high-dose arm. While 59 patients per arm were required and 62 patients were randomized to the high-dose arm, only 48 patients received the full high-dose regimen. Although this might lead to an underestimation of the effect of high-dose chemotherapy, this also reflects clinical reality. Our primary endpoint, the neoadjuvant response index, might be considered a limitation by some. While the prognostic value of pCR for TNBC has been widely accepted, the prognostic value of the NRI remains to be definitively established. Nowadays, there are also many novel methods available for the assessment of homologous recombination deficiency in breast cancer. Most focus on the detection of a “genomic scar”, such as “mutational signature 3” and HRDetect^17,18^. These methods are similar to the BRCA1-like assay, although typically have been shown to be associated with both BRCA1 and BRCA2 deficiency. A second group of assays aims to measure the ability of cancer cells to repair DNA damage via homologous recombination more directly, by quantifying RAD51 foci formation^19,20^. Which of these approaches is best able to select tumors for treatments exploiting homologous recombination deficiency remains to be determined.

The relatively long accrual time of our study meant that novel treatment options for high-risk TNBC became available in the meantime. The OlympiA trial enrolled patients with a germline *BRCA1* or *BRCA2* mutation and HER2-negative disease who either did not have a pCR after neo-adjuvant chemotherapy or had high risk disease treated with adjuvant chemotherapy. It reported that adjuvant olaparib improves invasive disease free survival with a HR of 0.58 (95% CI 0.41–0.82)^21^. In a subgroup analysis, patients who had received carboplatin-containing chemotherapy, also derived benefit from the added olaparib (HR 0.77 (95% CI 0.49–1.21)). Also the KEYNOTE-522 trial recently presented its results, showing that adding pembrolizumab to carboplatin-containing chemotherapy improved event-free survival in early TNBC with a HR of 0.63 (95% CI 0.48–0.82) in the overall study population and with a HR of 0.84 (0.55–1.28) for patients with T3-4 tumors^22^. Of note, patients with stage IIIc disease, which includes all patients with 10 or more positive lymph nodes (N3), were excluded in KEYNOTE-522. In our study, out of the 10 patients with N3 disease in the high-dose chemotherapy arm, none experienced a disease recurrence. Although these numbers are small, this finding is in line with the results of the N4+ trial which previously showed a benefit of adjuvant high-dose chemotherapy specifically for patients with 10 or more positive lymph nodes^23^. There is no data yet on the efficacy of immunotherapy in patients with early stage BRCA-altered breast cancer. However, in the TONIC trial, metastatic patients with BRCA1-like triple negative disease were less likely to respond to nivolumab treatment than patients with non-BRCA1-like disease^24^. It will be important to establish how these different treatment modalities (i.e. high-dose alkylating chemotherapy, conventional carboplatin, olaparib and pembrolizumab) can be utilized to ensure the best outcome for each TNBC patient. The SUBITO trial (NCT02810743), in which stage III, HER2-negative, BRCA-altered breast cancer patients are treated with 4 cycles of ddAC and then randomized between neoadjuvant carboplatin/paclitaxel plus adjuvant olaparib versus high-dose carboplatin/thiotepa/cyclophosphamide, aims to provide answers to some of these questions.

In this study, we observed substantially more short-term adverse events, including more fatigue, infections and nausea, in the high-dose arm compared to the conventional chemotherapy arm. This is in line with what has previously been reported for high-dose cyclophosphamide, thiotepa, carboplatin regimens given in the adjuvant or metastatic setting^25^. To our knowledge, this is the first report of a study giving high-dose chemotherapy in the neo-adjuvant setting. In this setting, it is important to establish that the side effects of high-dose chemotherapy do not cause unacceptable delays in surgery. We observed that the median time between the start of the last chemotherapy course and surgery was 7 days longer in the high-dose compared to the conventional arm. This difference is unlikely to affect the risk of disease recurrence. In addition, we observed a small numerical difference in the number of patients with complications related to surgery or radiotherapy (2/33 [6%] in the conventional arm vs 4/39 [10%] in the high-dose arm). This indicates that high-dose chemotherapy is possibly associated with a very limited increase in complications. Although no increased frequency of second primary tumors was observed in the high-dose arm, longer follow-up is needed to reliably assess the effect of treatment on the development of second primary tumors. Recently, the 20-year follow-up analysis of the N4+ trial assessed the long-term toxicities and found similar rates of second primary cancers, but more hypertension, hypercholesterolemia and dysrhythmias in the high-dose compared to the control arm. However, this did not translate in a higher incidence of major cardiovascular events^23^. An analysis of the same trial found that directly after treatment, quality of life had decreased more in the high-dose arm compared to the conventional arm. However, after one year both groups had reached the levels of healthy women again^26^. A smaller study looking at cognitive performance reported that patients treated with high-dose chemotherapy more often experienced a deterioration of cognitive performance six months after treatment compared to conventional chemotherapy^27^. It is unknown if these effects persist over time. The SUBITO study aims to further assess the evolution of patient reported outcomes and cognitive performance during long-term follow-up. Nonetheless, it is clear that the toxicities related to high-dose chemotherapy represent a substantial burden for patients, which is also reflected in in the relatively high proportion of patients with BRCA-altered tumors (35%) declining randomization in this study. Moreover, for patients with stage I-II N0 TNBC recent data on the prognostic and predictive significance of stromal tumor infiltrating lymphocytes (sTILs) suggest that further treatment de-intensification utilizing less or no (neo)adjuvant chemotherapy might be optimal for selected patients^28–30^.

In conclusion, we report that neoadjuvant high-dose alkylating chemotherapy does not improve NRI scores or pCR rates in patients with BRCA-altered TNBC. The study was underpowered for the secondary endpoints RFS and OS, and confounded by several patients receiving additional adjuvant chemotherapy after surgery. Exploratory analyses suggest that these non-significant trends may be driven by patients with stage III disease with a significant interaction between disease stage and treatment. While stage III patients seem to derive benefit from high-dose chemotherapy, stage II patients do not. Given recent publications on several promising novel treatments in early TNBC, establishing the optimal treatment strategy for stage IIIa-c, BRCA-altered TNBC patient remains an important challenge.

## Methods

### Study design and participants

NeoTN is a randomized, open-label, multicenter clinical trial, conducted in 13 hospitals in the Netherlands. Patients aged 18-60 years were eligible if they had histologically-confirmed TNBC (<10% ER-positive tumor cell nuclei; HER2 IHC 0/1+, or 2+ with no in situ hybridization-based amplification). Patients with an ER-negative but PR-positive tumor (>10% positive tumor cell nuclei), were only eligible when the ER-negativity was confirmed by the reference pathology lab at the Netherlands Cancer Institute. The patient had to have a tumor >20 mm (measured with MRI or ultrasound) and/or have axillary lymph node metastasis, confirmed by fine needle aspiration. Distant metastases had to be excluded by routine staging examinations. No prior chemotherapy or radiotherapy was allowed. No prior malignancy except for carcinoma in situ was allowed, unless treated ≥5 years ago with curative intent. All patients had to have a WHO performance-status 0-1. Adequate organ functions were required.

The study protocol (M09TNM) was approved by the Medical Ethics Committee of the Netherlands Cancer Institute. All patients signed written informed consent. Trial conduct and reporting followed Good Clinical Practice guidelines, the Declaration of Helsinki and the Consolidated Standards of Reporting Trials (CONSORT) reporting guideline.

### Randomization and masking

For all patients a tumor biopsy for BRAC1-like testing and a contrast enhanced (CE) MRI was obtained before start of chemotherapy. All patients received three courses of neoadjuvant dose-dense doxorubicin (60 mg/m^2^) and cyclophosphamide (600 mg/m^2^) (ddAC) given in a 2-weekly schedule with pegfilgrastim support. Meanwhile, tumor biopsies were tested for BRCA1-like status according to the methods described below.

In this manuscript, we will report on the patients with an BRCA-altered tumor, who were offered randomization between conventional and high-dose chemotherapy. For the full study design including a separate randomization for patients with non-BRCA-altered tumors see Supplementary Fig. 6. Randomization was done using minimization^31^ based on the following factors: initial MRI-based T‐stage (T1-2 vs T3-4), nodal‐status (negative vs positive), and age (<50 vs ≥50). Randomization was done by computer and once by dice in presence of two independent observers because of a technology failure. The trial was open label for patients and investigators.

### Procedures

*High-dose chemotherapy arm*. The high dose regimen consisted of a fourth ddAC course followed by Peripheral Blood Progenitor Cell (PBPC) harvest. Subsequently, two cycles of intermediate-dose alkylating therapy (miniCTC, Carboplatin 800 mg/m^2^, Thiotepa 250 mg/m^2^, and Cyclophosphamide 3000 mg/m^2^) were given, each followed by PBPC-reinfusion.

*Conventional chemotherapy arm*. Initially, conventional chemotherapy was guided by contrast-enhanced (CE)-MRI-response after three ddAC courses. A CE-MRI-based favorable response was defined as a change in tumor size measure by the maximum wash-out area diameter ≥25%^32^. If patients had a favorable response, they received three additional ddAC courses. In case of a non-favorable response, patients received non-cross-resistant chemotherapy: Three courses of capecitabine with docetaxel (CD). Capecitabine (800 mg/m^2^) twice daily on days 1 through 12 and docetaxel (75 mg/m^2^) on day 1, every three weeks. The trial was amended in June 2012, as new data became available showing increased pathological complete response (pCR) rates in TNBC when adding carboplatin^14,15^. After this amendment, all patients randomized to the conventional arm received three courses of carboplatin/paclitaxel (CP) after the initial three ddAC courses, independent of MRI-response. Carboplatin was given AUC = 6 on day 1 and paclitaxel (80 mg/m^2^) on days 1, 8 and 15, every three weeks.

After neoadjuvant chemotherapy completion, patients received adequate locoregional treatment, including surgery, and if indicated by local guidelines, radiotherapy. Adjuvant chemotherapy could be given if indicated by the treating physician.

Investigators graded AEs according to the NCI Common Toxicity Criteria v4.0, and judged relatedness of AEs to study medication. Only AEs grade ≥2 were recorded in the study database. As this is the first neoadjuvant study with high-dose chemotherapy in breast cancer, a research nurse manually checked the patient files of the hospital that contributed most patients to the neoTN study (the Netherlands Cancer Institute) for delays and complications related to surgery and radiotherapy in both treatment arms.

### Assessment of BRCA-altered status

Tumors were considered BRCA-altered when any of the following criteria applied: 1) a tumor in a known *BRCA1* or *BRCA2* germline mutation carrier; 2) a positive BRCA1-like test using either array comparative genomic hybridization (aCGH)^8^ or a multiplex ligation-dependent probe amplification (MLPA)-based assay on primary tumor tissue^33,34^; 3) If the BRCA1-like test could not be performed, a positive MLPA-test for *BRCA1* promoter hypermethylation using a methylation‐specific MLPA kit according to the manufacturer’s protocol (ME001B, MRC‐Holland, The Netherlands) on primary tumor tissue. Even if not required for eligibility, BRCA1-like and *BRCA1* promoter hypermethylation testing was performed if sufficient material was available. During follow-up information on germline mutations was updated and mutation status for genes other than *BRCA1* or *BRCA2* (e.g. *PALB2*) was collected. All information on patients’ germline mutation status came from tests conducted in accredited Dutch Clinical Genetics Departments in the context of standard clinical care.

### Endpoints

The primary endpoint for the comparison of conventional and high-dose neoadjuvant treatment was the neoadjuvant response index (NRI). The NRI is a score between 0 and 1 indicating the extent of tumor downstaging in breast and axilla after neoadjuvant treatment. Patients with a pCR get a score of 1, while partial responses get a score between 0 and 1, proportional to the response in tumor and lymph nodes^11^. An NRI of 0.7 was used as predefined cut-off^11^. Secondary endpoints included recurrence-free survival (RFS) and overall survival (OS). RFS was defined in line with the STEEP definition^35^ as time from randomization until locoregional recurrence, distant recurrence or death of any cause, whichever came first. Alive, recurrence-free patients were censored at time of last follow-up. OS was calculated from date of randomization to date of death with patients still alive censored at time of last follow-up. For analyses assessing the prognostic value of NRI or pCR, RFS is calculated from surgery (RFS^surgery^).

### Statistical analysis

Sample size was calculated based on the comparison between conventional and high-dose chemotherapy to show superiority in terms of the NRI. Assuming that NRI is not normally distributed, a Wilcoxon (Mann-Whitney) rank-sum test with a 2-sided significance level of 0.05 had 80% power to detect an improvement of an average NRI from 0.62 to 0.80 when 59 patients in each treatment arm were included.

In accordance with the test used for the sample size calculation, the two groups were compared on NRI using a Wilcoxon rank-sum test. RFS and OS were analyzed using the Kaplan-Meier method. Univariable and multivariable (subgroup) analyses on RFS and OS were performed using Cox proportional hazards regression. All tests were two-sided with a significance level of 0.05. The analyses by stage and pCR status were unplanned and exploratory in nature and were not corrected for multiple comparison. All outcome analyses were performed on the ITT population and patients were analyzed as randomized. For adverse events (AEs), patients were analyzed as treated (actual treatment received). Analyses were performed in SAS, v9.4 (SAS Institute) and R, v4.0.3.

### Reporting summary

Further information on research design is available in the Nature Research Reporting Summary linked to this article.

### Supplementary information

Supplementary Material

Reporting Summary

## Data Availability

The data collected for this study can be made available to others in de-identified form in the presence of a data transfer agreement. Requests for data sharing can be made to the corresponding author.
