# Supplementary material for: High-dose alkylating chemotherapy in BRCA-altered triple-negative breast cancer: the randomized phase III NeoTN trial
Source: NPJ Breast Cancer. 2023 Sep 9;9:75. doi: 10.1038/s41523-023-00580-9 (PMC10492793; doi:10.1038/s41523-023-00580-9)

Contents

Supplementary Table 1. BRCA1-like, germline mutation and BRCA1 promoter methylation 2

Supplementary Table 2. Mutual exclusivity germline mutation and BRCA1 promoter methylation 3

Supplementary Table 3. Extended patient characteristics for all patients with BRCA-altered tumors 4

Supplementary Table 4. Surgery and radiotherapy related complication in the patients treated at the Netherlands Cancer Institute 6

Supplementary Figure 1. Recurrence-free survival by stage 7

Supplementary Figure 2. Recurrence-free survival by type of conventional chemotherapy stratified by stage 8

Supplementary Figure 3. Recurrence-Free Survival by stage and pCR status 9

Supplementary Figure 4. Overall Survival by stage 10

Supplementary Figure 5. Overall survival after distant recurrence 11

Supplementary Figure 6. Study Design 12

Supplementary Table 1. BRCA1-like, germline mutation and BRCA1 promoter methylation

		BRCA1-like testing		
		**BRCA1-like**	**Sporadic-like**	**NA**
Total		104 (100%)	3 (100%)	14 (100%)
Germline mutation				
	BRCA1	13 (13%)	1 (33%)	9 (64%)
	BRCA2	1 (1%)	0 (0%)	1 (7%)
	PALB2	1 (1%)	0 (0%)	0 (0%)
	**No** [1]	61 (59%)	2 (67%)	3 (21%)
	**NA**	28 (27%)	0 (0%)	1 (7%)
BRCA1 promoter hypermethylation				
	**Yes**	24 (23%)	2 (67%)	4 (29%)
	**No**	42 (40%)	0 (0%)	2 (14%)
	**NA**	38 (37%)	1 (33%)	8 (57%)

[1] No mutation detected using the standard technique at the time (including at least BRCA1 and BRCA2 testing). NA = not assessed

Supplementary Table 2. Mutual exclusivity germline mutation and BRCA1 promoter methylation

		Germline mutation				
		**BRCA1**	**BRCA2**	**PALB2**	**No [1]**	**NA**
Total		24 (100%)	2 (100%)	1 (100%)	66 (100%)	29 (100%)
BRCA1 promoter hypermethylation						
	**Yes**	0 (0%)	0 (0%)	0 (0%)	24 (36%)	6 (21%)
	**No**	11	0 (0%)	0 (0%)	27 (41%)	7 (24%)
	**NA**	13	2 (100%)	1 (100%)	15 (23%)	16 (55%)

[1] No mutation detected using the standard technique at the time (including at least BRCA1 and BRCA2 testing). NA = not assessed

Supplementary Table 3. Extended patient characteristics for all patients with BRCA-altered tumors

	ddAC-only n=25 (42%)	ddAC-CD n=7 (12%)	ddAC-CP n=28 (47%)	All Conventional Chemotherapy n= 60 (%)	High-dose chemotherapy n= 62(%)	All randomized patients n= 122 (%)
Median age (IQR)	40 (35-53)	44 (38-48)	44 (38-50)	42 (37-51)	43 (36-50)	43 (37-51)
Menopausal status						
Pre	16 (64%)	6 (86%)	19 (68%)	41 (68%)	43 (70%)	84 (69%)
Peri	1 (4%)	0 (0%)	4 (14%)	5 (8%)	6 (10%)	11 (9%)
Post	8 (32%)	1 (14%)	5 (18%)	14 (23%)	12 (20%)	26 (21%)
NA	0	0	0	0	1	1
Size (T)						
1	2 (8%)	0 (0%)	2 (7%)	4 (7%)	4 (6%)	8 (7%)
2	17 (68%)	6 (86%)	21 (75%)	44 (73%)	47 (76%)	91 (75%)
3	4 (16%)	1 (14%)	5 (18%)	10 (17%)	10 (16%)	20 (16%)
4	2 (8%)	0 (0%)	0 (0%)	2 (3%)	1 (2%)	3 (2%)
Nodal status (N)						
0	9 (36%)	5 (71%)	12 (43%)	26 (43%)	21 (34%)	47 (39%)
1	10 (40%)	1 (14%)	9 (32%)	20 (33%)	26 (42%)	46 (38%)
2	2 (8%)	0 (0%)	2 (7%)	4 (7%)	5 (8%)	9 (7%)
3	4 (16%)	1 (14%)	5 (18%)	10 (17%)	10 (16%)	20 (16%)
Stage						
II	15 (60%)	6 (86%)	21 (75%)	42 (70%)	44 (71%)	86 (70%)
III	10 (40%)	1 (14%)	7 (25%)	18 (30%)	17 (27%)	35 (29%)
IV	0 (0%)	0 (0%)	0 (0%)	0 (0%)	1 (2%)	1 (1%)
Grade						
Well differentiated	0 (0%)	0 (0%)	0 (0%)	0 (0%)	1 (2%)	1 (1%)
Moderately differentiated	1 (7%)	0 (0%)	2 (11%)	3 (8%)	10 (20%)	13 (15%)
Poorly differentiated	13 (93%)	5 (100%)	16 (89%)	34 (92%)	38 (78%)	72 (84%)
NA	11	2	10	23	13	36

ER negative	25 (100%)	7 (100%)	28 (100%)	60 (100%)	62 (100%)	122 (100%)
0%	17 (89%)	4 (100%)	17 (74%)	38 (83%)	46 (98%)	84 (90%)
1-10%	2 (11%)	0 (0%)	6 (26%)	8 (17%)	1 (2%)	9 (10%)
Not specified	6	3	5	14	15	29
HER2						
0	12 (48%)	2 (33%)	14 (61%)	28 (52%)	38 (66%)	66 (59%)
1+	10 (40%)	2 (33%)	8 (35%)	20 (37%)	15 (26%)	35 (31%)
2+	3 (12%)	2 (33%)	1 (4%)	6 (11%)	5 (9%)	11 (10%)
3+	0 (0%)	0 (0%)	0 (0%)	0 (0%)	0 (0%)	0 (0%)
NA	0	1	5	6	4	10
MRI response						
Favorable	25 (100%)	0 (0%)	25 (89%)	50 (83%)	52 (87%)	102 (84%)
Non-favorable	0 (0%)	7 (100%)	3 (11%)	10 (17%)	8 (13%)	18 (15%)
NA	0	0	0	0	2	2
Adjuvant chemotherapy[1]						
Taxanes	5 (20%)	2 (29%)	1 (4%)	8 (13%)	2 (3%)	10 (8%)
Carboplatin	3 (12%)	2 (29%)	1 (4%)	6 (10%)	1 (2%)	7 (6%)
Capecitabine	0 (0%)	0 (0%)	0 (0%)	0 (0%)	1 (2%)	1 (1%)
AC	0 (0%)	0 (0%)	1 (4%)	1 (2%)	0 (0%)	1 (1%)
Other	0 (0%)	1 (14%)	0 (0%)	1 (2%)	0 (0%)	1 (1%)
Any	5 (20%)	3 (43%)	2 (7%)	10 (17%)	3 (5%)[2]	13 (11%)

[1] All patients who received adjuvant chemotherapy had not reached a pCR. [2] Of the 3 patients in the high-dose arm who received adjuvant chemotherapy, only one had actually received high-dose chemotherapy, while the other 2 received conventional chemotherapy because of patient preference or the patient’s condition being insufficient for high-dose treatment.

Supplementary Table 4. Surgery and radiotherapy related complication in the patients treated at the Netherlands Cancer Institute

Conventional chemotherapy (n=33)	
**patient**	**Complication (CTCAE grade)**
2	Seroma (grade 3)
129	Wound dehiscence (grade 2)
**High-dose chemotherapy (n=39)**	
**patient**	**Complication (CTCAE grade)**
169	Post-operative bleeding (grade 4)
1	Seroma (grade 3), Wound infection (grade 3)
143	(Radiation) pneumonitis (grade 2)
221	Wound infection (grade 4), Fever (grade 2)

72/122 (59%) patients received locoregional therapy at the Netherlands Cancer Institute

Supplementary Figure 1. Recurrence-free survival by stage

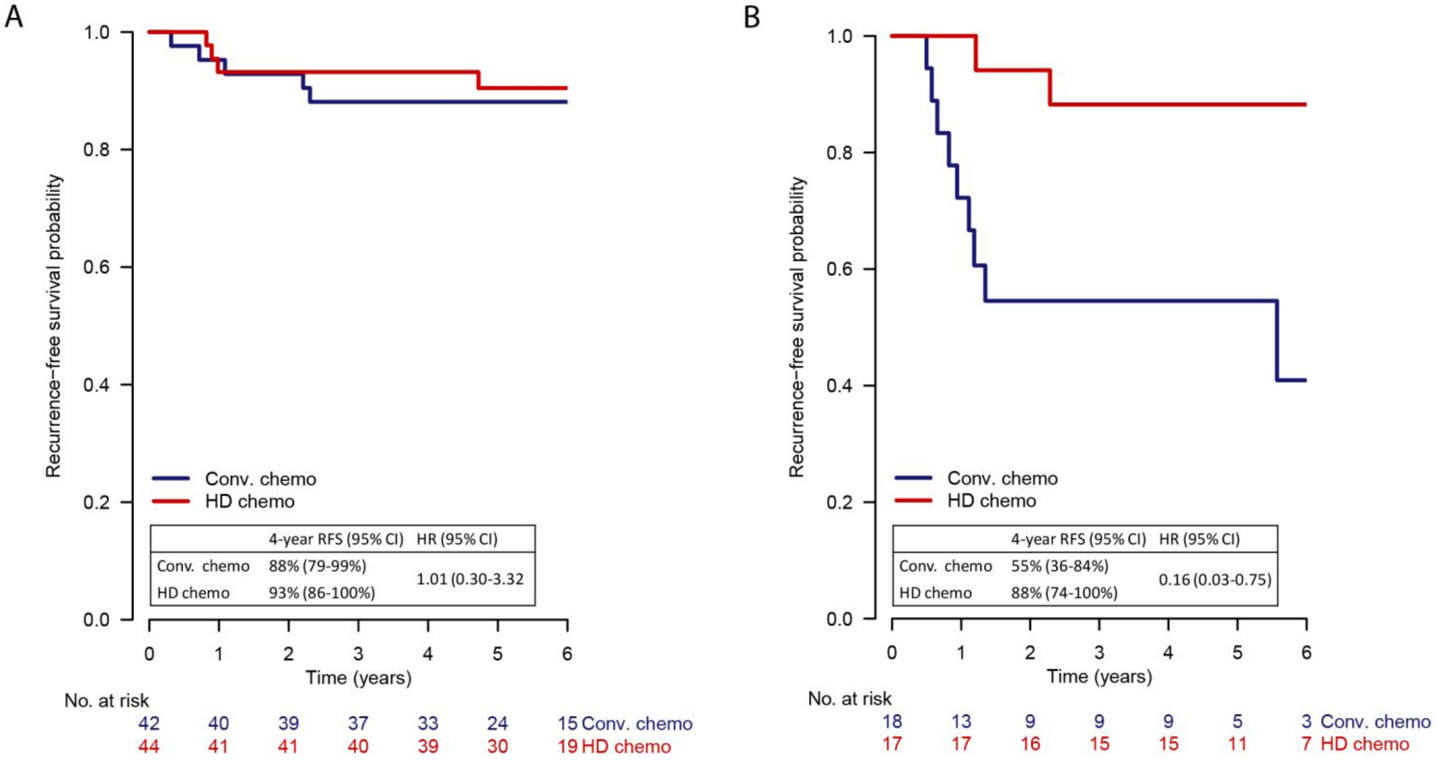

Recurrence-Free Survival for stage II patients (A) and stage III patients (B).

Supplementary Figure 2. Recurrence-free survival by type of conventional chemotherapy stratified by stage

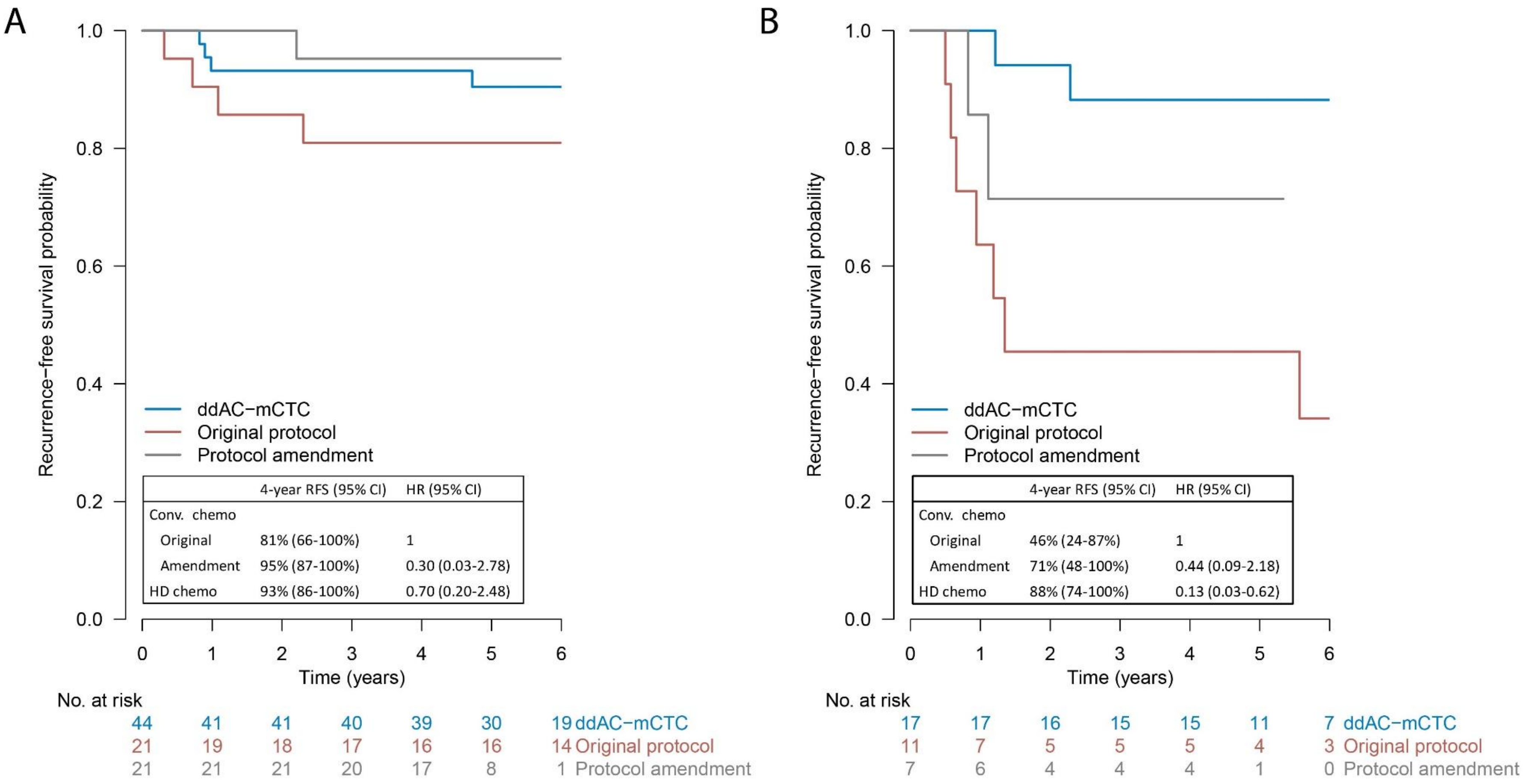

Recurrence-free survival for dose-dense chemotherapy (ddAC-mCTC) and convention chemotherapy according to the original protocol (3x ddAC followed by 3x ddAC in case of a MRI response or 3x CD in case of no response) or amended protocol (3x ddAC followed by CP) for patients with (A) stage 2 disease and (B) stage 3 disease.

Supplementary Figure 3. Recurrence-Free Survival by stage and pCR status

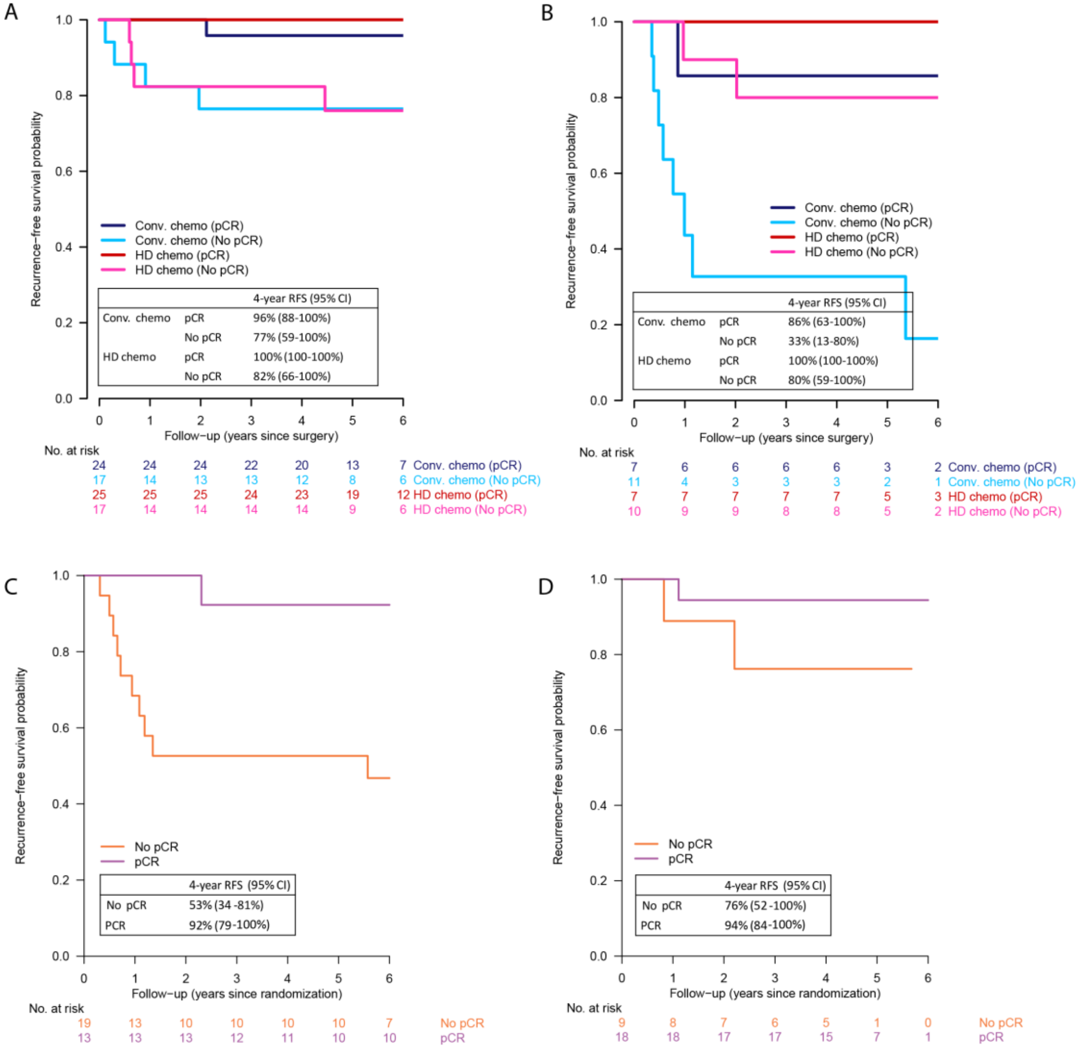

RFS^{surgery} (calculated from surgery) by pathological complete response (pCR) status and treatment arm for (A) stage II patients and (B) stage III patients and for patients treated in the conventional arm under the original protocol with ddAC or ddAC-CD (C) and under the amended protocol with ddAC-CP (D).

Supplementary Figure 4. Overall Survival by stage

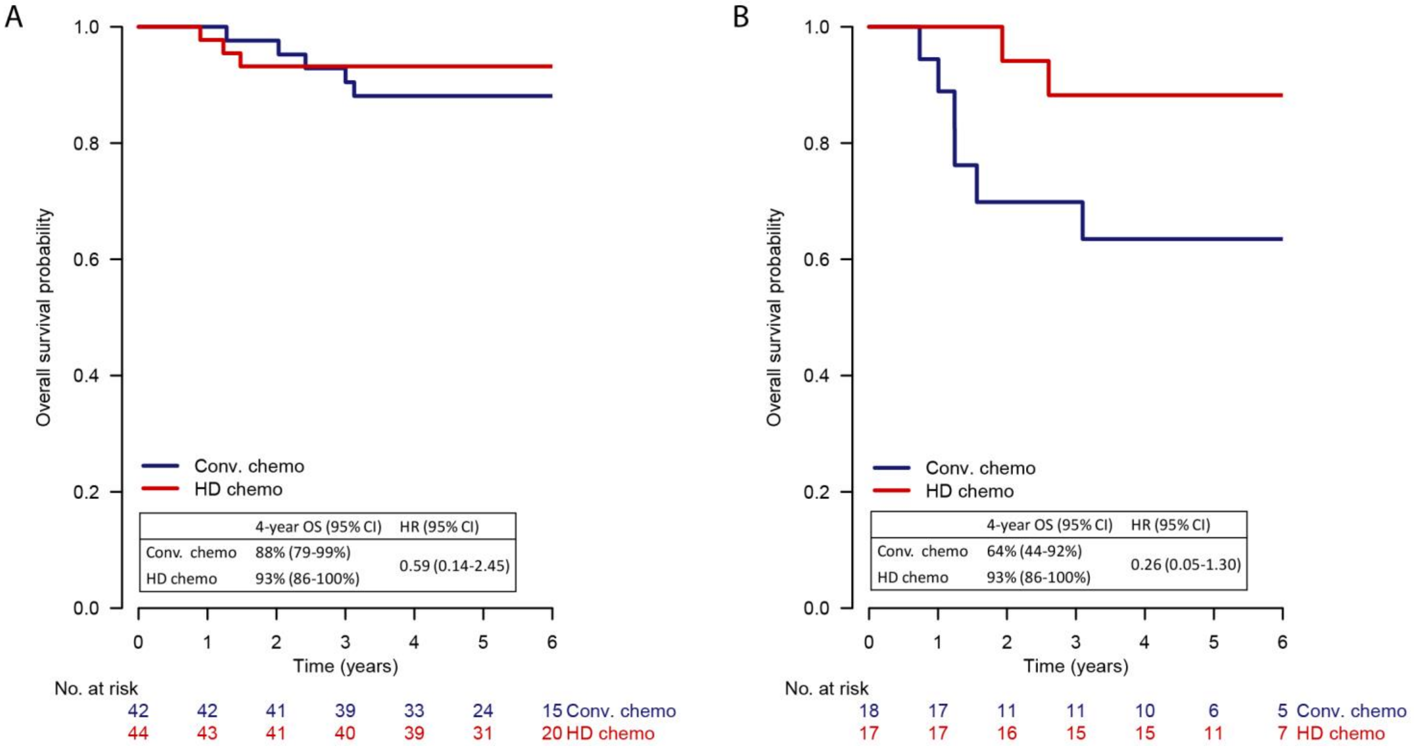

Overall Survival by treatment arm for stage II patients (A) and stage III patients (B). One patient in the intention to treat population who had stage IV disease at the time of diagnosis was left out of the analyses stratified by stage.

Supplementary Figure 5. Overall survival after distant recurrence

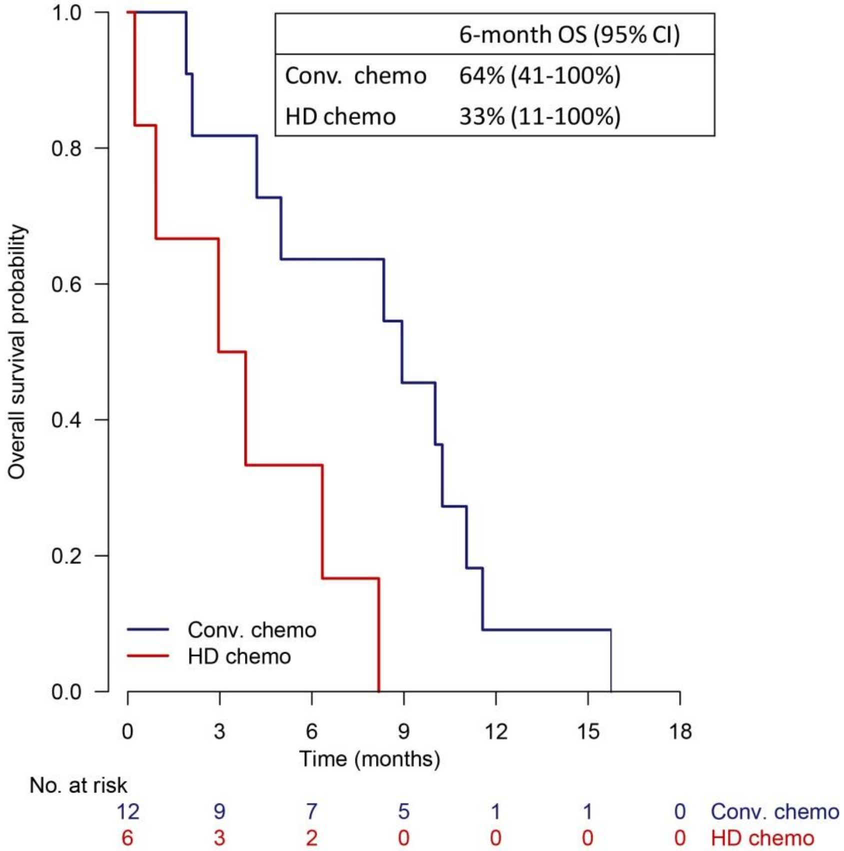

Overall survival calculated from first diagnosis of distant recurrence by treatment arm.

Supplementary Figure 6. Study Design

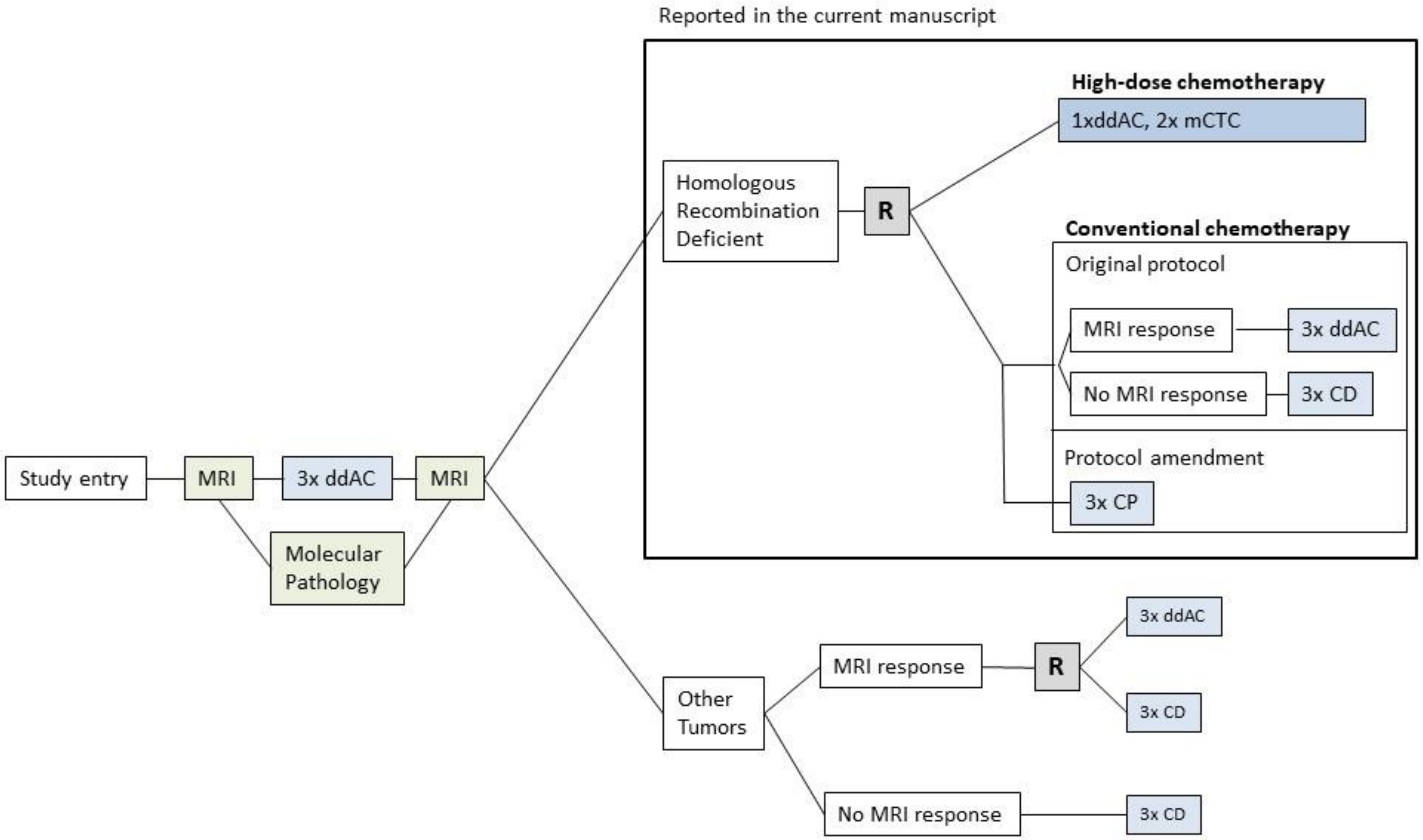

Supplement: Supplementary file 1 — Supplementary Material [file 41523_2023_580_MOESM1_ESM.pdf]
