# Supplementary material for: High-dose alkylating chemotherapy in BRCA-altered triple-negative breast cancer: the randomized phase III NeoTN trial
Source: NPJ Breast Cancer. 2023 Sep 9;9:75. doi: 10.1038/s41523-023-00580-9 (PMC10492793; doi:10.1038/s41523-023-00580-9)
Supplement: Supplementary file 2 — Reporting Summary [file 41523_2023_580_MOESM2_ESM.pdf]

nature portfolio

Corresponding author(s): Sabine Linn

Last updated by author(s): Aug 22, 2023

Reporting Summary

Nature Portfolio wishes to improve the reproducibility of the work that we publish. This form provides structure for consistency and transparency in reporting. For further information on Nature Portfolio policies, see our Editorial Policies and the Editorial Policy Checklist.

Statistics

For all statistical analyses, confirm that the following items are present in the figure legend, table legend, main text, or Methods section.

n/a	Confirmed	
☐	☒	The exact sample size (*n*) for each experimental group/condition, given as a discrete number and unit of measurement
☒	☐	A statement on whether measurements were taken from distinct samples or whether the same sample was measured repeatedly
☐	☒	The statistical test(s) used AND whether they are one- or two-sided<br>*Only common tests should be described solely by name; describe more complex techniques in the Methods section.*
☐	☒	A description of all covariates tested
☐	☒	A description of any assumptions or corrections, such as tests of normality and adjustment for multiple comparisons
☐	☒	A full description of the statistical parameters including central tendency (e.g. means) or other basic estimates (e.g. regression coefficient) AND variation (e.g. standard deviation) or associated estimates of uncertainty (e.g. confidence intervals)
☒	☐	For null hypothesis testing, the test statistic (e.g. *F*, *t*, *r*) with confidence intervals, effect sizes, degrees of freedom and *P* value noted<br>*Give P values as exact values whenever suitable.*
☒	☐	For Bayesian analysis, information on the choice of priors and Markov chain Monte Carlo settings
☒	☐	For hierarchical and complex designs, identification of the appropriate level for tests and full reporting of outcomes
☒	☐	Estimates of effect sizes (e.g. Cohen's *d*, Pearson's *r*), indicating how they were calculated

Our web collection on statistics for biologists contains articles on many of the points above.

Software and code

Policy information about availability of computer code

Data collection	Data was collected in a Microsoft Access database (version 14.0, later updated to 15.0 and 16.0).
Data analysis	R version 4.2.1 (2022-06-23 ucrt), packages chron 2.3-61, Hmisc 5.0-1, lattice 0.20-45, readxl 1.4.2, rms 6.6-0, survival 3.3-1 backports 1.3.0, base64enc 0.1-3, caret 6.0-94, cellranger 1.1.0, checkmate 2.0.0, class 7.3-20, cli 3.6.1, cluster 2.1.3, codetools 0.2-18, colorspace 2.0-2, compiler 4.2.1, conquer 1.2.1, data.table 1.14.2, digest 0.6.28, dplyr 1.1.2, evaluate 0.21, fansi 0.5.0, fastmap 1.1.0, foreach 1.5.1, foreign 0.8-82, Formula 1.2-4, future 1.23.0, future.apply 1.8.1, generics 0.1.1, ggplot2 3.4.2, globals 0.14.0, glue 1.6.2, gower 0.2.2, grid 4.2.1, gridExtra 2.3, gtable 0.3.0, htmlTable 2.3.0, htmltools 0.5.5, htmlwidgets 1.6.2, httr 1.4.6, ipred 0.9-12, iterators 1.0.13, kableExtra 1.3.4, knitr 1.43, lava 1.7.2.1, lifecycle 1.0.3, listenv 0.8.0, lubridate 1.9.2, magrittr 2.0.3, MASS 7.3-57, Matrix 1.4-1, MatrixModels 0.5-0, matrixStats 0.61.0, ModelMetrics 1.2.2.2, multcomp 1.4-17, munsell 0.5.0, mvtnorm 1.1-3, nlme 3.1-157, nnet 7.3-17, parallel 4.2.1, parallelly 1.28.1, pillar 1.9.0, pkgcong 2.0.3, plyr 1.8.6, polspline 1.1.19, pROC 1.18.0, prodlim 2019.11.13, purrr 1.0.1, quantreg 5.86, R6 2.5.1, Rcpp 1.0.7, recipes 0.1.17, reshape2 1.4.4, rlang 1.1.0, rmarkdown 2.11, rpart 4.1.16, rstudioapi 0.14, rvest 1.0.3, sandwich 3.0-1, scales 1.2.1, SparseM 1.81, splines 4.2.1, stats4 4.2.1, stringi 1.7.5, stringr 1.5.0, svglite 2.1.0, systemfonts 1.0.4, TH.data 1.1-0, tibble 3.2.1, tidyselect 1.2.0, timechange 0.2.0, timeDate 3043.102, tools 4.2.1, utf8 1.2.2, vctrs

For manuscripts utilizing custom algorithms or software that are central to the research but not yet described in published literature, software must be made available to editors and reviewers. We strongly encourage code deposition in a community repository (e.g. GitHub). See the Nature Portfolio guidelines for submitting code & software for further information.

Data

Policy information about availability of data

All manuscripts must include a data availability statement. This statement should provide the following information, where applicable:

- Accession codes, unique identifiers, or web links for publicly available datasets
- A description of any restrictions on data availability
- For clinical datasets or third party data, please ensure that the statement adheres to our policy

The data collected for this study can be made available to others in de-identified form in the presence of a data transfer agreement. Requests for data sharing can be made to the corresponding author.

Research involving human participants, their data, or biological material

Policy information about studies with human participants or human data. See also policy information about sex, gender (identity/presentation), and sexual orientation and race, ethnicity and racism.

Reporting on sex and gender	No sex- or gender-based analyses were performed as the fast majority of patients with triple negative breast cancer, including those in this study, are female and identify as women.
Reporting on race, ethnicity, or other socially relevant groupings	This study does not report on race, ethnicity, or other socially relevant groupings
Population characteristics	Patients with stage II-III, histologically-confirmed, triple negative breast cancer, aged 18-60 years. No prior chemotherapy or radiotherapy was allowed. No prior malignancy except for carcinoma in situ was allowed, unless treated ≥5 years ago with curative intent. All patients had to have a WHO performance-status 0-1. Adequate organ functions were required.
Recruitment	Patients were recruited via medical oncologists in 13 hospitals in the Netherlands.
Ethics oversight	Medical Ethics Committee of the Netherlands Cancer Institute

Note that full information on the approval of the study protocol must also be provided in the manuscript.

Field-specific reporting

Please select the one below that is the best fit for your research. If you are not sure, read the appropriate sections before making your selection.

☒ Life sciences ☐ Behavioural & social sciences ☐ Ecological, evolutionary & environmental sciences

For a reference copy of the document with all sections, see nature.com/documents/nr-reporting-summary-flat.pdf

Life sciences study design

All studies must disclose on these points even when the disclosure is negative.

Sample size	Sample size was calculated based on the comparison between conventional and high-dose chemotherapy to show superiority in terms of the NRI. Assuming that NRI is not normally distributed, a Wilcoxon (Mann-Whitney) rank-sum test with a 2-sided significance level of 0.05 had 80% power to detect an improvement of an average NRI from 0.62 to 0.80 when 59 patients in each treatment arm were included.
Data exclusions	No patients were excluded from the intention to treat analysis, for the full overview of patient enrollment please see the CONSORT diagram included the manuscript
Replication	NA
Randomization	Randomization was done by computer and once by dice in presence of two independent observers because of a technology failure. The trial was open label for patients and investigators.
Blinding	Due to the nature of the high-dose chemotherapy treatment, blinding was not possible.

Reporting for specific materials, systems and methods

We require information from authors about some types of materials, experimental systems and methods used in many studies. Here, indicate whether each material, system or method listed is relevant to your study. If you are not sure if a list item applies to your research, read the appropriate section before selecting a response.

Materials & experimental systems

n/a	Involved in the study
☒	☐ Antibodies
☒	☐ Eukaryotic cell lines
☒	☐ Palaeontology and archaeology
☒	☐ Animals and other organisms
☐	☒ Clinical data
☒	☐ Dual use research of concern
☒	☐ Plants

Methods

n/a	Involved in the study
☒	☐ ChIP-seq
☒	☐ Flow cytometry
☒	☐ MRI-based neuroimaging

Clinical data

Policy information about clinical studies

All manuscripts should comply with the ICMJE guidelines for publication of clinical research and a completed CONSORT checklist must be included with all submissions.

Clinical trial registration	NCT01057069
Study protocol	Available as supplementary material for this manuscript
Data collection	Patients enrolled Between February 2010 and February 2016, follow-up was collected until March 2021
Outcomes	The primary endpoint for the comparison of conventional and high-dose neoadjuvant treatment was the neoadjuvant response index (NRI). The NRI is a score between 0 and 1 indicating the extent of tumor downstaging in breast and axilla after neoadjuvant treatment. Patients with a pCR get a score of 1, while partial responses get a score between 0 and 1, proportional to the response in tumor and lymph nodes.17 An NRI of 0.7 was used as predefined cut-off.17 Secondary endpoints included recurrence-free survival (RFS) and overall survival (OS). RFS was defined in line with the STEEP definition18 as time from randomization until locoregional recurrence, distant recurrence or death of any cause, whichever came first. Alive, recurrence-free patients were censored at time of last follow-up. OS was calculated from date of randomization to date of death with patients still alive censored at time of last follow-up. For analyses assessing the prognostic value of NRI or pCR, RFS is calculated from surgery (RFSsurgery).
